# Supplementary figures and images for: Effect of cow’s milk with different PUFA n-6: n-3 ratios on performance, serum lipid profile, and blood parameters of grower gilts
Source: PLoS One. 2022 May 26;17(5):e0258629. doi: 10.1371/journal.pone.0258629 (PMC9135250; doi:10.1371/journal.pone.0258629)

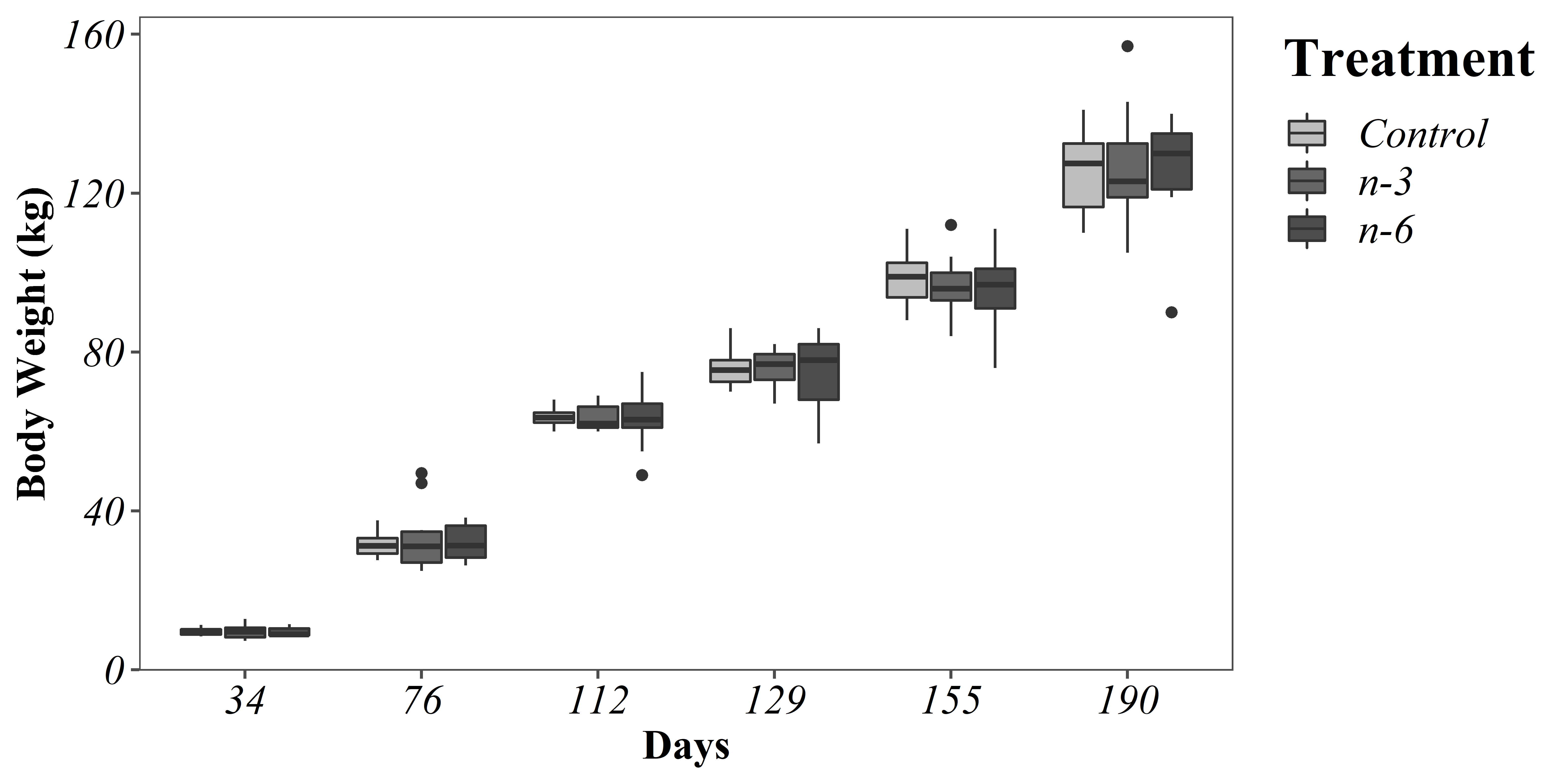

Supplement: S1 Fig — (TIFF) [file pone.0258629.s001.tiff]

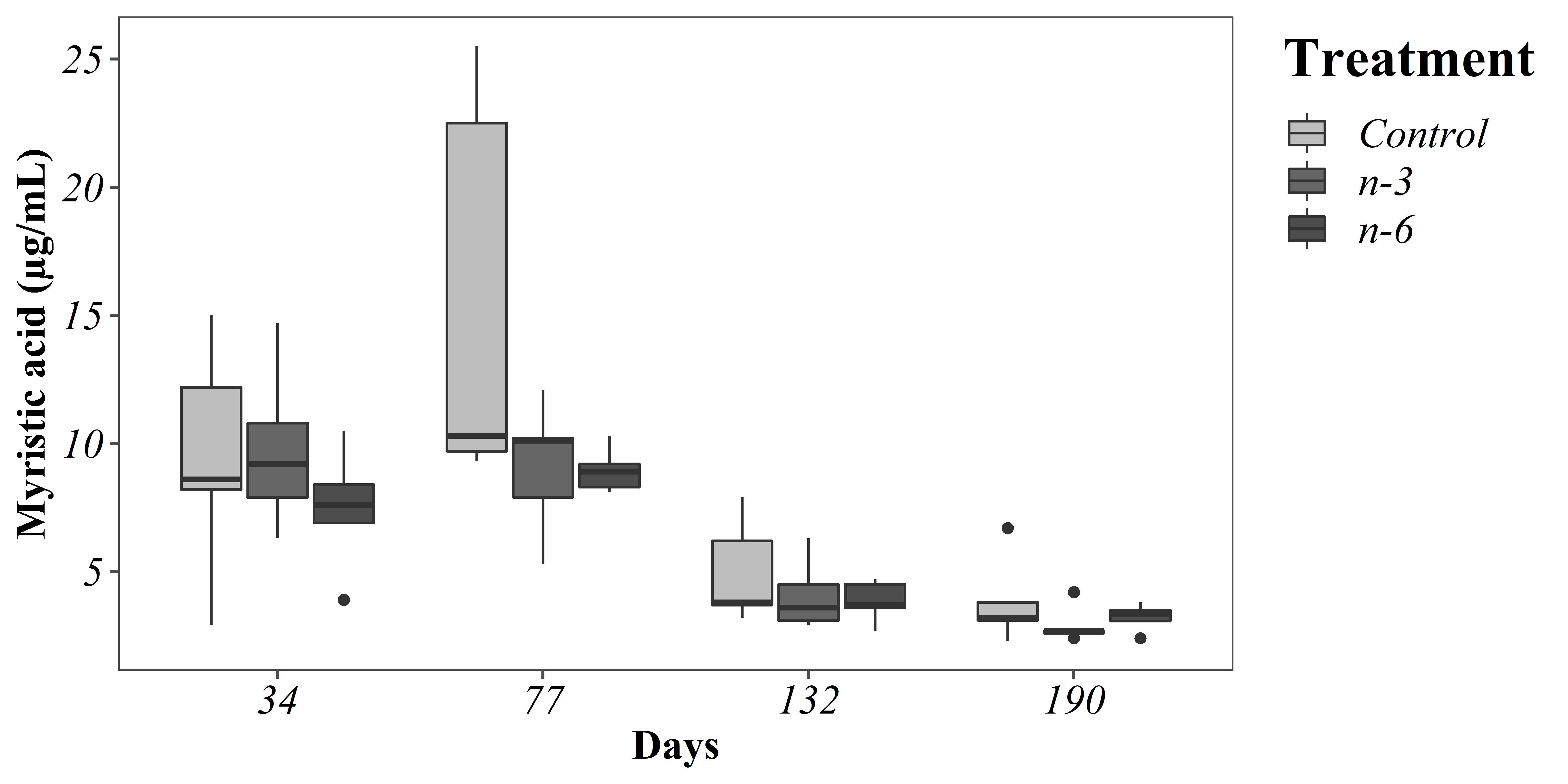

Supplement: S2 Fig — (TIFF) [file pone.0258629.s002.tiff]
