# Supplementary material for: Effect of cow’s milk with different PUFA n-6: n-3 ratios on performance, serum lipid profile, and blood parameters of grower gilts
Source: PLoS One. 2022 May 26;17(5):e0258629. doi: 10.1371/journal.pone.0258629 (PMC9135250; doi:10.1371/journal.pone.0258629)
Supplement: S1 Table — (PDF) [file pone.0258629.s003.pdf]

Effect of cow's milk with different PUFA n-6: n-3 ratios on performance, serum lipid profile, and blood parameters of grower gilts.

Authors: Leriana Garcia Reis[1], Thiago Henrique da Silva[1], Márcia Saladini Vieira Salles[2], André Furugen Cesar Andrade[3], Simone Maria Massami Kitamura Martins[1], Paula Lumy Takeuchi[4],Ana Maria Centola Vidal[5], Arlindo Saran Netto[1]

Running head: Milk with an altered lipid profile without impair the health

[1]Department of Animal Science, School of Animal Science and Food Engineering, University of São Paulo, Rua Duque de Caxias Norte, 225, 13635-900, Pirassununga, SP, Brazil.

[2]Animal Science Institute, Avenida Bandeirantes, 2419, Ribeirão Preto 14030-640, Brazil

[3]Department of Animal Reproduction, School of Veterinary and Animal Science, University of São Paulo, Avenida Duque de Caxias Norte, 225, Pirassununga 13635-900, Brazil.

[4]Department of Internal Medicine, Scholl of Medicine of Ribeirão Preto, University of São Paulo, Avenida Bandeirantes, 3900, Ribeirão Preto, 14049-900, Brazil.

[5]Department of Veterinary Medicine, Scholl of Animal Science and Food Engineering, University of São Paulo, Rua Duque de Caxias Norte, 225, 13635-900, Pirassununga, SP, Brazil.

Corresponding author: Arlindo Saran Netto. Phone number: +55 19 35654000/+55 19 35654039/+55 19 997270373. Email: saranetto@usp.br

Key words: Fatty acid metabolism, health, hemogram, polyunsaturated fatty acids, supplementation, vegetable oil.

Supplementary material

Table S1. Composition of the experimental diets of cows expressed on dry matter percentage

Item	Treatment[a]		
Ingredients, %	CC	SO	LO
Ground corn	30.5	28	28
Soybean meal	17	17	17
Soybean oil	-	2.5	-
Linseed oil	-	-	2.5
Urea	0.5	0.5	0.5
Limestone	0.3	0.3	0.3
Dicalcium phosphate	0.15	0.15	0.15
Salt	0.5	0.5	0.5
Mineral complex[b]	1.05	1.05	1.05
Corn silage	50	50	50
Chemical Composition, %			
Dry matter	92.01	92.61	91.68
Mineral matter	3.38	3.19	3.18
Crude protein	16.64	16.15	16.24
Neutral detergent fiber	29.97	29.48	29.42
Acid detergent fiber	18.73	18.14	17.99
Ether extract	3.49	5.67	5.78
Lignin	3.91	3.56	3.61
Total carbohydrate	75.95	74.01	74.18
Non-fiber carbohydrate	45.99	44.53	44.76
Total digestible nutrients	66.77	69.31	69.46

[a] CC = Control; SO = Soybean Oil; LO = Linseed Oil.

[b] Composition per kilogram of product: Sulfur (S) 80g, Magnesium (Mg) 20g, Potassium (K), 20g, Manganese (Mn) 1000mg, Zinc (Zn) 2500 mg, Copper (Cu) 1500 mg, Cobalt (Co) 100mg, Iodine (I) 80 mg, Selenium (Se) 20 mg, Calcium (Ca) 180g, Phosphorus (P) 90 g, Fluor (F) 300mg.
